# Molecular Cloning, Characterization, and Expression Analysis of an Estrogen Receptor-Related Receptor Homologue in the Cricket, *Teleogryllus emma*

**DOI:** 10.1673/031.010.18801

**Published:** 2010-11-01

**Authors:** Hui He, Gengsi Xi, Xiao Lu

**Affiliations:** College of Life Sciences, Shaanxi Normal University, Xi'an, 710062, People's Republic of China

**Keywords:** cDNA cloning, developmental expression

## Abstract

The estrogen receptor-related receptors (ERRs) are a group of nuclear receptors that were originally identified on the basis of sequence similarity to estrogen receptors. The three mammalian ERR genes have been implicated in diverse physiological processes ranging from placental development to maintenance of bone density, but the function and regulation of ERRs in invertebrates are not well understood. A homologue of human ERR was isolated from the cricket *Teleogryllus emma* (Ohmachi and Matsumura) (Orthoptera: Gryllidae). The full-length cDNA of *T. emma* ERR, termed TeERR, has 1618 base pair (bp) and contains a 5′?-untranslated region of 140 bp and a 3′?-untranslated region of 272 bp. The open reading frame of TeERR encodes a deduced 401 amino acid peptide with a predicted molecular mass of 45.75 kilodaltons. The results of sequence alignments indicate that the TeERR protein shares an overall identity of 65%–82% with other known ERR homologues, and is most closely related to that of *Nasonia vitripennis* (Hymenoptera: Pteromalidae) and *Apis mellifera* (Apidae). Real-time quantitative reverse transcription-polymerase chain reaction was performed to compare the TeERR mRNA expression level at the whole body and gonad during *T. emma* development. The data revealed that TeERR mRNA is differentially expressed during *T. emma* development, with the highest expression level in embryos and the lowest in the body of late-instar larvae. The levels of TeERR transcripts also varied throughout gonad development; interestingly testicles had higher higher expression levels than ovaries at every development stage. These results suggest that TeERR has potential significance in the regulation of development in *T. emma*, due to its expression during different developmental periods.

## Introduction

The estrogen receptor-related receptors (ERRs) are members of the nuclear receptor superfamily of transcription factors. Three ERR genes have been identified in mammals, ERRα? (NR3B1), ERRβ? (NR3B2), and ERRγ? (NR3B3) (Giguere et al. 1988; Chen et al. 1999; Hong et al. 1999; Heard et al. 2000). ERRs and estrogen receptors (ERs) have overlapping affinities for co-activators and DNA-binding sites, but differ markedly in ligand binding and activation (Vanacker et al. 1999a, 1999b; Giguere 2002). Unlike ERs, ERRs do not bind estradiol and have been reported to be either constitutively active (Hong et al. 1999; Xie et al. 1999; Greschik et al. 2002) or activated by an unidentified ligand (Vanacker et al. 1999b). While high-affinity ERR agonists have not been identified, some ER ligands, including 4-hydroxytamoxifen and diethylstilbestrol, can antagonize ERR activity (Coward et al. 2001; Tremblay et al. 2001a, 2001b).

The functions and target genes of ERRs are not yet well understood. ERRα? has been shown to interfere with estrogen (Giguere 2002) and other steroid signals (Catherine et al. 2008) and also to be involved in the regulation of energy metabolism. ERRα?-null mutant mice are essentially normal with reduced body weight and peripheral fat deposits, which supports the hypothesis that ERRα? helps to regulate energetic metabolism and fat storage (Luo et al. 2003). The roles of the other two closely related paralogs (ERRβ? and ERRγ?) are still poorly understood. In contrast to the mild ERRα? knockout phenotype, ERRβ?-null mutants die during development due to defects in placental formation (Luo et al. 1997). The role of ERRγ? has not been elucidated through knockout experiments, but high expression has been noted in differentiating neural tissues (Hermans et al. 2000).

Although ERR homologues have been identified in a wide variety of mammals, they are still not well documented in invertebrates, especially insects. Only one ERR homolog gene was identified in the fruit fly, *Drosophila melanogaster* (Laudet 1997), and the ant, *Polyrhachis vicina* (Ouyang 2009). Genebank deposits predicted the ERR gene sequence of *Nasonia vitripennis, Apis mellifera, D. melanogaster, Anopheles stephensi, Aedes aegypti,* and *Culex quinquefasciatus*.

Field crickets can be important agricultural pests. They can also become household problems in late summer when they move out of fields and into buildings. In this study, an ERR homologue from *Teleogryllus emma* (Ohmachi and Matsumura) (Orthoptera: Gryllidae), which has been used as a model insect to study gene expression in the polyphagous insect (Kim et al. 2005; Kim et al. 2008), was cloned for the first time and named TeERR. TeERR mRNA expression patterns at the embryo, distinct developmental stages of the whole bodies and gonads of different sexes were studied by real-time quantitative reverse transcription-polymerase chain reaction (RT-PCR).

## Materials and Methods

### Insects

*T. emma* were obtained from a commercial supplier. This species typically undergoes six instar stages before reaching maturity after the observation of ecdysising. The crickets were raised at 26 ±± 1°° C, 60% RH, and a photoperiod of 12:12 L:D. Approximately 50 individuals were reared together in glass Containers (35 cm ×× 25 cm ×× 12 cm) that were covered with mosquito screening mesh to allow air circulation. The bottom of each container was lined with 3 cm of dry sand and pieces of folded cardboard to provide refuge. *T. emma* were fed goldfish flakes and fresh lettuce every two days. Water was provided via Petri dishes filled with wet cotton wool that were replaced also every two days. Embryos, larvae, and adults, when prepared for extracting total RNA, were collected and immersed in liquid nitrogen and stored at -80°° C until used.

### Molecular cloning of TeERR

Total RNA was extracted from pooled samples of ten frozen *T. emma* adult heads ( randomly selected) by means of a procedure with TAKARA RNAiso Plus and then immediately reverse transcribed for the generation of cDNA using a first strand cDNA synthesis kit with an oligo(dT) primer (Fermentas Life Science, www.frementas.com) following the manufacturer's instructions.

**Table 1.**
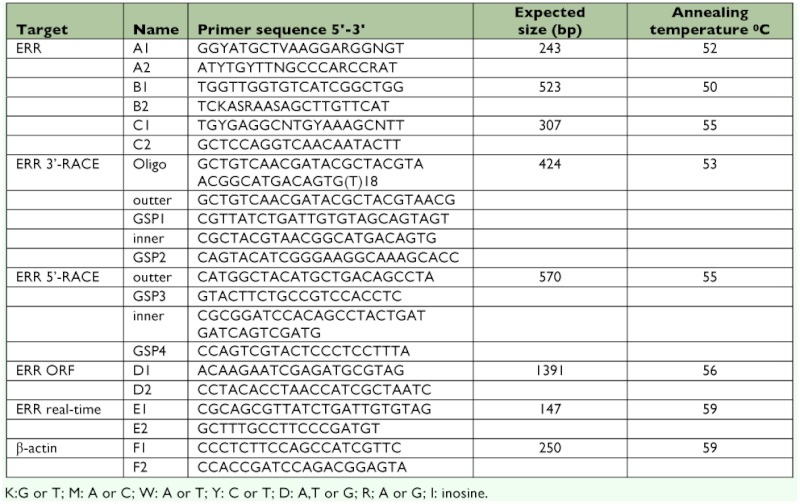
Oligonucleotide primers used for cDNA cloning and real-time quantitative reverse transcription polymerase chain reaction

Three fragments of TeERR cDNA was amplified by nested PCR using degenerated primers (Table 1) designed on the basis of the conserved motifs of published ERR homologues from other insect species (*D. melanogaster; A. mellifera; N. vitripennis; A. stephensi; Ae. aegypti* and *Cx. quinquefasciatus*). Inosine was used to reduce primer mismatch. PCR amplification was performed in 50 μ?l reaction volumes with the following protocol: 94°° C for 3 min; followed by 35 cycles of 94°° C for 45s, different melting temperatures for 45s, and 72°° C for 30s; and a final extension at 72°° C for 10 min. PCR products of the expected size were purified from agarose gel using a Gel Extraction Kit (Axygen, www.axygen.com) and subcloned into the pMD19-T simple vector using a TA Cloning kit (Takara Bio Inc., www.takara-bio.com).Three PCRpositive colonies were selected for sequencing.

### 3′? and 5′? rapid amplification of cDNA ends

The full-length cDNA of TeERR was amplified by 3′? and 5′? rapid amplification of cDNA ends (RACE) using 3′? Full RACE Core Set and 5′? Full RACE Core Set (Takara Bio Inc.), following the manufacturer's instruction. The first-strand cDNA for 3′?RACE was obtained through the reaction of reverse transcriptase M-MLV (RNase H) (Takara Bio Inc.) with heat-denatured total RNA and an oligo(dT) adaptor primer. The cDNA was amplified with the gene-specific primer (GSP1) (Table 1) and 3-RACE outer primer. Nested PCR was performed with the gene-specific primer (GSP2) (Table 1) and 3′?RACE inner primer. 5′? rapid amplification of cDNA ends is the same as 3′?-RACE using the two pairs of gene-specific primers (GSP3, 4) (Table 1). PCR reactions were performed as described above except for different templates and annealing temperatures (Table 1).

The PCR products from 3′?-RACE and 5′?RACE were cloned into a TA cloning vector (Takara Bio Inc) for sequencing Using the primers designed from the 3′?-RACE and 5′?RACE products (D1 and D2, Table 1), the full-coding cDNA was amplified by RT-PCR and sequenced to confirm the coding region of TeERR.

### Structure and phylogenetic analysis of TeERR

The open reading frame (ORF) of TeERR was searched using the National Center for Biotechnology Information ORF Finder (http//www.ncbi.nlm.nih.gov/gorf/gorf.html). Signal peptide prediction was performed using the SignalP program (Centre for Biological Sequence Analysis; www.cbs.dtu.dk./services/SignalP/) (Bendtsen et al. 2004). Potential functional motifs of the protein sequence were analyzed using the PROSITE database (Expert Protein Analysis System; Swiss Institute of Bioinformatics; http://myhits.isb-sib.ch/cgi-bin/motif_scan). The identities of TeERR protein to known ERR protein sequences were analyzed using MegAlign (DNASTAR package). Sequence alignments based on the amino acid sequences of known ERRs were preformed with Clustal X 1.81 (Jeanmougin et al. 1998) followed by manual inspection. From these alignments, phylogenetic trees were constructed using the Neighbor-Joining method with a bootstrap test of 2000 replicates and a Poisson correction model implemented in MEGA version 4.0 (Tamura et al. 2006).

### Real-time quantitative RT-PCR

Quantitative RT-PCR was performed to quantify the relative levels of expression of TeERR transcripts at the whole bodies and gonads between sexes during development. Total RNAs were isolated from a pooled sample of 10 individual embryos and different development stages of larvae, adults, and gonads dissected from males and females. Five μ?g of total RNAs was used for reverse transcription using the method mentioned above, with the addition that the RNAs for RT-PCR were treated with RNase-free DNase 1 (Roche Applied Science, www.rocheapplied-science.com/index.jsp) to prevent genomic DNA contamination. To compensate for difference in loading/RT efficiency, β?-actin was used as the endogenous control. Primer (E1,2) of TeERR, and primers (F1,2) of β?actin were used in real-time PT-PCR (Table 1), which resulted in PCR products of 145 and 250 bp, respectively. Reactions were performed using the iQ5 apparatus (Bio-Rad Laboratories Inc., www.bio-rad.com) with a SYBR Premix Ex Taq Kit (Takara Bio Inc.), and the detailed protocol was as follows: 95°° C for 1 min, 40 cycles of 95°° C for 10 s and 59°° C for 25 s, followed by a dissociationcurve program from 55 to 95°° C with a heating rate of 0.5°° C every step and continuous-fluorescence acquisition.

One of the cDNA samples was used to construct standard curves for TeERR and β? actin after serial dilution and after the slopes of the curves were obtained. Expression levels were determined using the formula *F* = 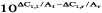 proposed by Zhang et al 2005), where *F* is the relative expression of the samples, *C_t_* is the number of cycles necessary to reach a defined fluorescence threshold, *A*_t_ and *A*_r_ are the slopes of the TeERR and β? -actin standard curves, respectively, and Δ? *C*_t.t_ and Δ? *C*_t.r_ denote the difference between the *C_t_* value of samples and the calibrator for TeERR and β? -actin, respectively. The *F* value of the calibrator was designated as 1. The normalized amount (TeERR/β?-actin) was deduced from the C_t.t_ and C_t.r_ of the calibrator sample to obtain the difference between Δ? *C*_t.t_/*A*_t_ and Δ? *C*_t.r_/*A*_r_. Here the cDNA of embryos was selected as the calibrator in analyzing TeERR expression at different development stages of the whole body. For analyzing TeERR expression in the gonads, the gonads of a fourth instar male were selected as the calibrator. Measurements were performed in triplicate using the pooled samples. Date was entered into an Excel spreadsheet (Microsoft Corp., www.microsoft.com) to obtain *F* values. Analysis of the data was carried out using SPSS 13.1 (SPSS Inc. 2004).

**Figure 1.**
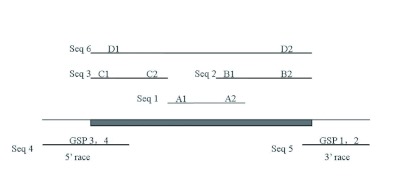
Cloning strategy and map of the primers used for amplifying the full sequence of the TeERR gene: degenerated primers A1, A2 for sequence 1 of 243 bp; degenerated primers B1.B2 for sequence 2 of 523 bp; degenerated primers C1.C2 for sequence 3 of 424 bp; Four specific primers GSP 1,2,3,4 were used in 3'RACE (424 bp) and 5'RACE (570 bp). D1,D2 for confirming the open reading frame of the TeERR. High quality figures are available online.

## Results

### Cloning and characterization of TeERR cDNA

An ERR cDNA fragment was isolated firstly by RT-PCR using total RNA extracted from adult *T. emma* and degenerate primers (Table 1) located in the two conserved regions of nuclear receptors: the DNA binding domain (DBD) and the ligand binding domain (LBD). The full-length cDNA of TeERR was amplified by 5′?-RACE and 3′?-RACE based on the scheme shown in Figure 1. 3′?-RACE product of 343 bp and 5′?-RACE product of 570 bp were obtained from nested PCR with the gene-specific primers. Then the overlapping cDNA sequences obtained were assembled with ContigExpress software (Invitrogen, www.invitrogen.com) and the full-length TeERR cDNA of 1618 bp was obtained. To confirm this TeERR cDNA sequence, RT-PCR with the gene-specific primers D1, 2 was performed. A product of 1391 bp spanning the entire ORF was obtained, and its complete overlap with the assembled TeERR cDNA was verified. TeERR cDNA (Genebank accession No. FJ770332) was 1618 bp in length and contained an ORF of 1206 bp with a 5′?untranslated region of 140 nucleotides followed by an initiating ATG codon. Stop codon TAA was at position 1347–1349.

**Figure 2.**
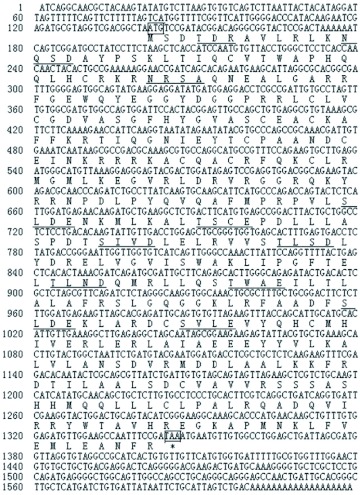
The cDNA and deduced amino acid sequence of TeERR from *Teleogryllus emma*. The sequence was 1618 bp long and encoded a protein of 401 amino acid residues. The initiating codon ATG and stop codon TAA are in box. A series of function motifs in the TeERR protein sequence are underlined, details in the text. High quality figures are available online.

The full-length TeERR cDNA and deduced amino acid sequences are shown in Figure 2. The TeERR protein consists of 401 amino acid residues with a calculated molecular mass of 45.75 kilodaltons (kDa) and an isoelectric point of 9.14. Analysis with the SignalP program and GlycoMod tools showed that there was neither an N-terminal signal sequence nor N-linked oligosaccharide motif in this protein, suggesting that the TeERR protein may be an unglycosylated or nonsecreted protein. A series of predicted function motifs were found in the TeERR protein sequence using the PROSITE program, including two N-glycosylation sites, ASN-GLYCOSYLATION at 13–16 (NQSD) and 41–44 (NRSA); nine casein kinase 11 phosphorylation sites, CK2-PHOSPHO-SITE at 2-5 (SDTD), 173–176 (SLDE), 184–187 (TSCE), 198–201 (SIVD), 209–212 (TLSD), 235–238 (TLND), 246–249 (TWAE), 273–276 (SLDE), and 283–286 (SVLE); two Nmyristoylation sites, MYRISTYL at 61–66 (GGYDGG), 84–89 (GVASCE); one protein kinase C phosphorylation site, PKCPHOSPHO-SITE at 4–6 (TDR); two Tyrosine kinase phosphorylation sites, TYRPHOSPHO-SITE at 52–59 (RRFgEWQY) and 154–161(RRNpDLPY); one nuclear hormone receptors DNA-binding domain profile, NUCLEAR-REC-DBD at 68–143; one Zinc finger C4 type (two domains), zf-C4 (69–144); one ligand-binding domain of nuclear hormone receptor, Hormone-recep at 215–395.

### Alignment analysis and phylogenetic-tree construction

**Figure 3.**
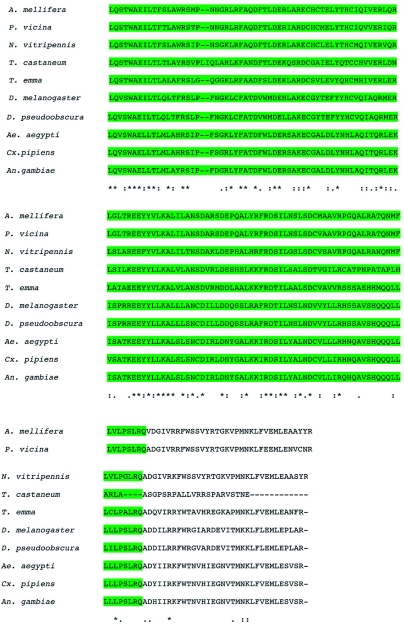
Alignment of the ERR homologue sequences for several insects species, including *Apis mellifera, Phylloxiphia vicina, Nasonia vitripennis, Tetropium castaneum, Teleogryllus emma, Drosophila melanogaster, Drosophila pseudoobscura, Aedes aegypti, Culex pipiens*, and *Anopheles gambiae* using the ClustalX method. Asterisk (*) indicates the same amino acids; and ““.”” shows the degree of conservation of different amino acids; Green shading shows the conserved domain ZnF_C4 and Hol 1 of the ERRs; and yellow shading indicates the difference in the hinge region of TeERR and other ERR homologs. High quality figures are available online.

A multiple alignment of the deduced animo acid sequence of TeERR with other known ERR homologues was performed with Clustal X 1.81 and modified by BOXSHADE (European Molecular Biology Network, www.ch.embnet.org/software/BOX_form.html). Comparison of the translated cDNA sequence with those of known nuclear receptors showed most similarity to the estrogen related receptor (ERR) from the flies, *N. vitripennis* (68% identity), followed by the beetle, *A. mellifera* and the ant, *Phylloxiphia vicina* (67–68% identity). By alignment of the cricket receptor sequence with those of ERRs from other insects (Figure 2), a five-domain structure characteristic of all members of the steroid receptor superfamily (Green and Chambon 1988) could be identified: an A/B domain (activation region; amino acids 1–66), a C domain (DNA binding; 67–139), a D domain (hinge, 140–211), an E domain (ligand binding, 212–368) and a very short F domain (369–401). There was high sequence conservation with the other ERRs in the DNA- and ligand-binding domains, some conservation within the D domain, and very little in the A/B domain (Figure 3). The deduced amino acid sequence less closely resembled other nuclear receptor coding sequences. The cDNA clone was therefore designated as the *T. emma* estrogen related receptor (TeERR).

**Figure 4.**
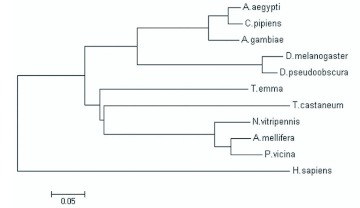
Phylogenetic tree constructed on the basis of alignment of the animo acid sequences of ERR homologues and showing the evolutionary relationship of TeERR with other insect species (*Apis mellifera, Phylloxiphia vicina, Nasonia vitripennis, Tetropium castaneum, Teleogryllus emma, Drosophila melanogaster, Drosophila pseudoobscura, Aedes aegypti, Culex pipiens*, and *Anopheles gambiae*) of the ERR family with *Homo sapiens* used as the outgroup. Numbers at branch nodes are percentages of bootstrap confidence values derived from 2000 replications. High quality figures are available online.

The phylogenetic trees including known homologs of ERR protein sequences of *T. emma* (GenBank accession no. FJ770332), *A. mellifera* (GenBank accession no. XP_392385), *P. vicina* (GenBank accession no. EF_474463), *N. vitripennis* (GenBank accession no. XP_00160403 3), *T. castaneum* (GenBank accession no. XP_001862599), *D. melanogaster* (GenBank accession no. NP_729340), *D. pseudoobscura* (GenBank accession no. XP_001354210), *Ae. aegypti* (GenBank accession no. EAT34188), *C. pipiens* (GenBank accession no. XP_001862 599), and *A. gambiae* (GenBank accession no. XP_321343), were constructed using the neighbor-joining method with Poisson correction (Figure 4), with *Homo sapiens* (GenBank accession no. NP_004443) used as the outgroup. Results from the phylogenetic trees revealed that TeERR shared about 68% identity with *A. mellifera* ERR, *N. vitripennis* ERR, and *P. vicina* ERR; 56–61% identity with *D. melanogaster* ERR, *Ae. aegypti* ERR, *C. pipiens* ERR, and *A. gambiae* ERR; and 50% identity with human ERR.

**Figure:**
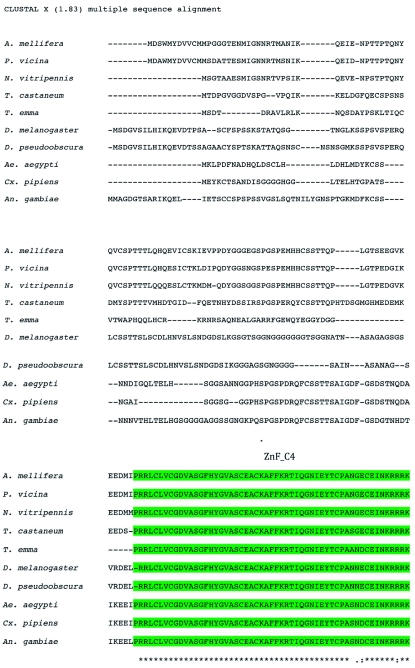


**Figure:**
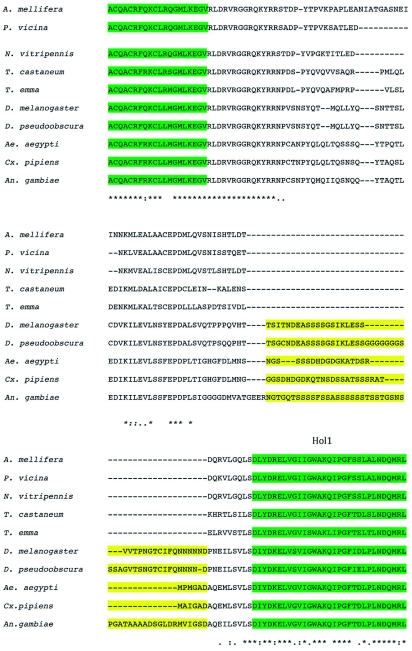


### Analysis of TeERR mRNA expression

**Figure 5.**
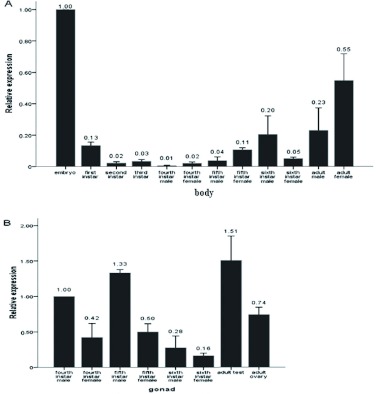
Relative expression profiles (mean ±± 2 SEM, *n* = 3) of the TeERR gene, determined by real-time PCR. A: TeERR mRNA at the whole body during different developmental stages. All expression levels are shown relative to the expression level in the embryo. B: TeERR mRNA at gonad during different developmental stages. All expression levels are shown relative to the expression level in fourth instar male. High quality figures are available online.

Levels of expression of TeERR mRNA at the whole body and gonad of different developmental stages were detected by means of quantitative RT-PCR. Previous studies clearly indicated that the analyzed β? -actin mRNA levels remain fairly constant in tissues of insects regardless of their developmental or physiological condition (Claeys et al. 2003; Simonet et al. 2004). Consequently, the β? actin gene was selected as the endogenous control in RT-PCR analysis of *T. emma*. To obtain precise quantification, the specific PCR products and the absence of primer-dimers were confirmed by viewing the single peak in the melting curve of the genes (TeERR and β?actin) tested (unpublished data). TeERR relative expression levels were calculated using the formula *F* = 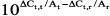 for each replicate. The results displayed that TeERR was expressed in all samples at different levels in both the bodies and gonads (Figure 5). In the whole body of different developmental stages, the TeERR gene was found to be expressed at the highest level in the embryo, then significantly less in the larvae, and least in fourth instar; later the expression is slightly increased in the adult. In the gonad of different developmental stages, the TeERR gene was found to be expressed at the highest level in adult male testicle and lowest in sixth instar female ovary, but there was no statistical difference between them. Interestingly, the level of expression in the testicle was always higher than the ovary at each developmental stage of the gonad (Figure 5B).

## Discussion

In this study, a full-length cDNA sequence of a unique ERR gene from the cricket *T. emma* was isolated and characterized. The cDNA sequence of TeERR and its deduced amino acid sequence reflected a high degree of homology with the ERR homologues identified from other animals, indicating that this newly isolated cDNA encoded the cricket *T. emma* ERR protein. The TeERR gene shared about 68% identity in amino acid sequence with Hymenoptera ERRs. The ERRs of dipteran insects were all in the 56–61% range and had 50% identity with human ERRs, which indicated that the ERR gene is conserved in evolutionary pathways. Given this comparison, it could be suggested that the phylogenetic relationship of TeERR was closer to human ERRs than the DERR was to human ERRs. This was similar to the results of *A. mellifera* genes for circadian rhythm, RNA interference, and DNA methylation (Honey Bee Genome Sequencing Consortium 2006). The results of multiple alignments showed that the DBD and the LBD of the ERR proteins were highly conserved and that these regions might include the main functional domains. The predicted functional motifs were mainly located in these domains, such as the protein kinase C phosphorylation site, the tyrosine kinase phosphorylation site etc. These data indicated that ERR protein was involved in signal transduction, which might be conserved during long-term evolution.

Phylogenetic and structural studies have proposed that ERRs are closely related to the ERs among the steroid hormone receptor (NR3) subfamily (Escriva et al. 2000). However, this view has been challenged by recent analysis suggesting that ERRs are equally related to ERs and SRs (Thornton et al. 2003). In the present study, only one ERR was cloned from the cricket *T. emma*. This was the same for other invertebrates such as *D. melanogaster, Ciona intestinalis* (a tunicate), and *Branchiostoma floridae* (amphioxus, an ephalochordate) (Sullivan and Thummel 2003; Bardet et al. 2005; Park et al. 2008) that also had a single ERR gene. In contrast, no ERR homolog has been recognized in the *Caenorhabditis elegans* genome (Sluder et al. 2001). During the invertebrate to vertebrate transition, the single *ERR* gene underwent at least two waves of duplication, which should theoretically lead to four genes in vertebrates. Three genes were identified in mammals, but four or five genes were identified in some fish (Bardet et al. 2004). The evolutionary history of *ERR* genes was therefore not clearly defined. This research indicated that the invertebrate single *ERR* might be an ancestral form of vertebrate *ERRs*.

The different levels of TeERR mRNA expression at different developmental stages in the whole body were measured in this experiment. The TeERR gene was found to be expressed at the highest level in the embryo, then significantly decreased in the larvae and then increased a small amount in the adult. The gene expression pattern in earlier stages was similar to that of not only *Drosophila* ERR (Sullivan et al. 2003) but also sea urchin (Howard et al. 2006) and ascidian ERR (Imai et al. 2004). Knockdown of ERRa expression using morpholino antisense oligonucleotides in zebrafish indicated a novel role for ERR in regulating morphogenic movement during gastrulation (Bardet et al. 2005a). The highest expression level in embryo indicated that the ERR mRNA in *T. emma* during embryogenesis might play an important role in cell proliferation and differentiation. In larvae, expression was significantly decreased. Morphogenesis occurred during the transition from embryo to larva, and the major regulative function of ERR in embryo might be lower in the larva. More research is needed to determine the effect of ERR in embryos in later developmental stages. In mice, ERRα? and ERRγ? are broadly expressed in adult tissues (Bonnelye et al. 1997a; Heard et al. 2000; Hermans et al. 2000). In contrast, ERRβ? had more limited expression. The four ERRs showed distinct, partially overlapping mRNA expression patterns in adult tissues of Atlantic killifish, *Fundulus heteroclitus* (Tarrant et al. 2006). A unique ERR gene from *T. emma* whose function may coincide with other animal ERRs was found in this study. The abundant expression of TeERR in adults suggest a broad expression pattern as in mammalian ERRs. Our goal now is to describe the spatial patterns of ERR-transcript expression in adult *T. emma* cricket tissues.

From a physiological point of view, estrogens have been implicated in a variety of processes, such as the homeostasis of bone and female reproductive organs. Interestingly, ERRα? has been suggested to play an active role in bone physiology (Bonnelye et al. 2001). Mammalian ERRs are expressed in reproductive tissues (amongst others) both during development and in the adult. These observations suggest functional interconnections between estrogen-signaling and ERR activities, which remain to be demonstrated *in vivo* (Giguere 2002). In this study, the TeERR mRNA expression was detected in the gonad of different developmental stages. The expression level in the testicle was always higher than in the ovary at each developmental stage, which might be related to some functions of ERR in *T. emma* reproduction. In insects, ecdysone is a counterpart of estrogens in mammals (Ouyang 2009), so this research suggested that TeERR might interfere or collaborate with ecdysone signaling. The sequence of the ecdysone receptor response element (EcRE) was similar to an estrogen receptor response element (ERE) as they all had a ““GGTCA”” motif. The estrogen- related receptor response element (ERRE) possessed ““TnAAGGTCA”” as well as a ““GGTCA”” motif and an ERR bound to ERRE (Martinez et al. 1991). ERR might combine with EcRE and participate in ecdysone signaling to modulate the reproduction of *T. emma*. More research is needed to determine the precise role of ERR in insects.

In summary, this study described, for the first time, a unique ERR homologue cloned from the cricket *T. emma*. The finding of differential expression of the TeERR gene at distinct developmental stages of the body and gonad may indicate the physiological importance of TeERR in cricket. The relationship of ERR and ecdysone should be further investigated.

## Abbreviations

**ER**,: estrogen receptors;
**ERR**,: estrogen receptor-related receptors;
**TeERR**,: *Teleogryllus emma* estrogen receptor-related receptors
